# Supplementary material for: Resolving the full spectrum of human genome variation using Linked-Reads
Source: Genome Res. 2019 Apr;29(4):635–45. doi: 10.1101/gr.234443.118 (PMC6442396; doi:10.1101/gr.234443.118)
Supplement: Supplemental Material [file supp_gr.234443.118_Supplemental_longranger-2.2.zip › longranger-2.2.tar.bz2!/lr2.2-public/lib/go/src/code.google.com/vitess/docs/user-guide/production-planning.html]

Vitess / Production Planning


Toggle navigation

- Overview
- Guides
- Reference
- Blog
- GitHub

- #### Overview

  - What is Vitess
  - Scaling MySQL with Vitess
  - Key Concepts
- #### Getting Started

  - Run Vitess on Kubernetes
    - Custom Docker Build
  - Run Vitess Locally
- #### User Guide

  - Introduction- Client Libraries- Backing Up Data- Reparenting
        - Schema Management
        - VSchema Guide
        - Schema Swap (Tutorial)
        - Sharding
          - Horizontal Sharding (Tutorial, automated)
          - Horizontal Sharding (Tutorial, manual)
          - Sharding in Kubernetes (Tutorial, automated)
          - Sharding in Kubernetes (Tutorial, manual)
        - Vitess Sequences
        - MySQL Server Protocol
        - Vitess and Replication
        - Update Stream
        - Row Based Replication
        - Topology Service
        - Transport Security Model
        - Launching
          - Scalability Philosophy
          - Production Planning
          - Server Configuration
          - 2PC Guide
          - Troubleshooting
        - Upgrading
- #### Reference Guides

  - Vitess API- vtctl Commands
- #### Other Resources

  - Presentations- Blog- Roadmap
- #### Contributing

  - Contributing to Vitess- GitHub Workflow- Code Reviews
- GitHub

# Production Planning

- #### Overview

  - What is Vitess
  - Scaling MySQL with Vitess
  - Key Concepts
- #### Getting Started

  - Run Vitess on Kubernetes
    - Custom Docker Build
  - Run Vitess Locally
- #### User Guide

  - Introduction- Client Libraries- Backing Up Data- Reparenting
        - Schema Management
        - VSchema Guide
        - Schema Swap (Tutorial)
        - Sharding
          - Horizontal Sharding (Tutorial, automated)
          - Horizontal Sharding (Tutorial, manual)
          - Sharding in Kubernetes (Tutorial, automated)
          - Sharding in Kubernetes (Tutorial, manual)
        - Vitess Sequences
        - MySQL Server Protocol
        - Vitess and Replication
        - Update Stream
        - Row Based Replication
        - Topology Service
        - Transport Security Model
        - Launching
          - Scalability Philosophy
          - Production Planning
          - Server Configuration
          - 2PC Guide
          - Troubleshooting
        - Upgrading
- #### Reference Guides

  - Vitess API- vtctl Commands
- #### Other Resources

  - Presentations- Blog- Roadmap
- #### Contributing

  - Contributing to Vitess- GitHub Workflow- Code Reviews

## Provisioning

### Estimating total resources

Although Vitess helps you scale indefinitely, the various layers do consume CPU and memory. Currently, the cost of Vitess servers is dominated by the RPC framework which we use: gRPC (gRPC is a relatively young product). So, Vitess servers are expected to get more efficient over time as there are improvements in gRPC as well as the Go runtime. For now, you can use the following rules of thumb to budget resources for Vitess:

Every MySQL instance that serves traffic requires one VTTablet, which is in turn expected to consume an equal amount of CPU. So, if MySQL consumes 8 CPUs, VTTablet is likely going to consume another 8.

The memory consumed by VTTablet depends on QPS and result size, but you can start off with the rule of thumb of requesting 1 GB/CPU.

As for VTGate, double the total number of CPUs you’ve allocated for VTTablet. That should be approximately how much the VTGates are expected to consume. In terms of memory, you should again budget about 1 GB/CPU (needs verification).

Vitess servers will use disk space for their logs. A smoothly running server should create very little log spam. However, log files can grow big very quickly if there are too many errors. It will be wise to run a log purger daemon if you’re concerned about filling up disk.

Vitess servers are also likely to add about 2 ms of round-trip latency per MySQL call. This may result in some hidden costs that may or may not be negligible. On the app side, if a significant time is spent making database calls, then you may have to run additional threads or workers to compensate for the delay, which may result in additional memory requirements.

The client driver CPU usage may be different from a normal MySQL driver. That may require you to allocate more CPU per app thread.

On the server side, this could result in longer running transactions, which could weigh down MySQL.

With the above numbers as starting point, the next step will be to set up benchmarks that generate production representative load. If you cannot afford this luxury, you may have to go into production with some over-provisioning, just in case.

### Mapping topology to hardware

The different Vitess components have different resource requirements e.g. vtgate requires little disk in comparison to vttablet. Therefore, the components should be mapped to different machine classes for optimal resource usage. If you’re using a cluster manager (such as Kubernetes), the automatic scheduler will do this for you. Otherwise, you have to allocate physical machines and plan out how you’re going to map servers onto them.

Machine classes needed:

#### MySQL + vttablet

You’ll need database-class machines that are likely to have SSDs, and enough RAM to fit the MySQL working set in buffer cache. Make sure that there will be sufficient CPU left for VTTablet to run on them.

The VTTablet provisioning will be dictated by the MySQL instances they run against. However, soon after launch, it’s recommended to shard these instances to a data size of 100-300 GB. This should also typically reduce the per-MySQL CPU usage to around 2-4 CPUS depending on the load pattern.

#### VTGate

For VTGates, you’ll need a class of machines that would be CPU heavy, but may be light on memory usage, and should require normal hard disks, for binary and logs only.

It’s advisable to run more instances than there are machines. VTGates are happiest when they’re consuming between 2-4 CPUs. So, if your total requirement was 400 CPUs, and your VTGate class machine has 48 cores each, you’ll need about 10 such machines and you’ll be running about 10 VTGates per box.

You may have to add a few more app class machines to absorb any additional CPU and latency overheads.

## Lock service setup

The Lock Service should be running, and both the global and local instances
should be up. See the
Topology Service
document for more information.

Each lock service implementation supports a couple configuration command line
parameters, they need to be specified for each Vitess process.

For sizing purposes, the Vitess processes do not access the lock service very
much. Each *vtgate* process keeps a few watches on a few local nodes (VSchema
and SrvKeyspace). Each *vttablet* process will keep its on Tablet record up to
date, but it usually doesn't change. The *vtctld* process will access it a lot
more, but only on demand to display web pages.

As mentioned previously, if the setup is only in one cell, the global and local
instances can be combined. Just use different top-level directories.

## Production testing

Before running Vitess in production, please make yourself comfortable first with the different operations. We recommend to go through the following scenarios on a non-production system.

Here is a short list of all the basic workflows Vitess supports:

- Failover / Reparents
- Backup/Restore
- Schema Management / Schema Swap
- Resharding / Horizontal Resharding Tutorial
- Upgrading

Contact: vitess@googlegroups.com  **·**  
Announcements  **·**  
© 2017 Vitess powered by Google Inc
